# Cognitive control and unusual decisions about beauty: an fMRI study

**DOI:** 10.3389/fnhum.2014.00520

**Published:** 2014-07-21

**Authors:** Albert Flexas, Jaume Rosselló, Pedro de Miguel, Marcos Nadal, Enric Munar

**Affiliations:** ^1^Human Evolution and Cognition (EvoCog) Group, IFISC (UIB-CSIC), Psychology, Universitat de les Illes BalearsPalma, Spain; ^2^Clínica RotgerPalma, Spain; ^3^Department of Basic Psychological Research and Research Methods, University of ViennaVienna, Austria

**Keywords:** esthetic appreciation, art, functional magnetic resonance imaging, reaction time, conflict monitoring

## Abstract

Studies of visual esthetic preference have shown that people without art training generally prefer representational paintings to abstract paintings. This, however, is not always the case: preferences can sometimes go against this usual tendency. We aimed to explore this issue, investigating the relationship between “unusual responses” and reaction time in an esthetic appreciation task. Results of a behavioral experiment confirmed the trend for laypeople to consider as beautiful mostly representational stimuli and as not beautiful mostly abstract ones (“usual response”). Furthermore, when participants gave unusual responses, they needed longer time, especially when considering abstract stimuli as beautiful. We interpreted this longer time as greater involvement of cognitive mastering and evaluation processes during the unusual responses. Results of an fMRI experiment indicated that the anterior cingulate (ACC), orbitofrontal cortex (OFC) and insula were the main structures involved in this effect. We discuss the possible role of these areas in an esthetic appreciation task.

## Introduction

Decisions about the beauty of objects involve a variety of cognitive processes (Leder et al., 2004). Some of them are related to the perception of the stimulus and the retrieval of information stored in memory. Others have to do with analysing the object in terms of content and style, while others bring into play the viewers' interests and knowledge. Finally, the cognitive and affective states that result from the previous processes ground esthetic emotions and the esthetic judgments. As a result of such processes, laypeople —those lacking specific training in the arts—generally show a marked preference for representational stimuli, that is to say, those depicting clearly discernible objects. Vessel and Rubin (2010), for instance, created novel abstract stimuli and compared them to real-world images, finding that participants preferred the non-abstract stimuli. Cusack et al. (2010) studied the impact of visual art in patient waiting rooms, and also found that most of the patients preferred looking at landscape paintings. These results support other experiments involving visual esthetic preference (Hekkert and van Wieringen, 1996; Furnham and Walker, 2001b; Nadal et al., 2010; Pihko et al., 2011). Thus, there seems to be a general tendency to prefer representational stimuli to abstract stimuli.

The most widespread explanation for this tendency is based on the effects of familiarity on preference. Because laypeople are more familiar with representational art than abstract art (Zajonc, 1968; Furnham and Walker, 2001b; Cela-Conde et al., 2002), and familiarity tends to increase preference, they prefer the former to the latter. This explanation is supported by the fact that these differences disappear with expert participants (Hekkert and van Wieringen, 1996; Pihko et al., 2011), who are more familiar with abstract paintings than laypeople. Familiarity is also a recurrent explanation for positive assessments in other kinds of art (Hargreaves, 1984; Zizak and Reber, 2004). However, it is conceivable that the effect actually works in the opposite sense, that stimuli that are liked are perceived as more familiar, as reflected in Monin's (2003) *warm glow heuristic*. Nonetheless, some studies found that familiar stimuli are rated as less liked than unfamiliar stimuli (Cantor and Kubose, 1969; Faw and Nunnally, 1971). Tinio and Leder (2009) demonstrated that familiarization generates contrast effects for complexity: participants familiarized with simple stimuli judge complex stimuli as more beautiful and participants familiarized with complex stimuli judge simple stimuli as more beautiful.

Another possible explanation for laypeople's greater preference for representational over abstract art is that esthetic processing is based on the appraisal of valence of objects, a general cognitive process that applies to both non-art and art objects (Brown et al., 2011). Therefore, affectively meaningful objects in representational paintings are preferred over “meaningless” colors and forms. In a similar way, Vessel and Rubin (2010) suggest that visual preferences for real-world images are driven by the semantic content of stimuli.

Evidently, laypeople do not consider every representational stimulus to be beautiful and every abstract painting to be not beautiful. Occasionally, responses go against this general tendency. They consider some representational stimuli to be not beautiful and some abstract stimuli to be beautiful. Thus, our first purpose was to study these responses against the general tendency. As an initial approximation to this issue, we re-analyzed some data from Nadal et al. (2010). This analysis revealed that actually more abstract paintings were classified as ugly than beautiful, and that representational paintings were generally classified as beautiful. Furthermore, abstract paintings considered beautiful (response against the tendency) took on average 800 ms more than considering them as ugly (see supplementary material for the extended results).

The present study attempts to deepen our understanding of these responses against the general tendency depending on the kind of stimuli. First, we designed a behavioral experiment consisting of a visual esthetic preference task with two kinds of stimuli: abstract paintings on the one hand, and representational paintings and photographs on the other. The paradigm was similar to those used previously in neuroimaging studies (Cela-Conde et al., 2004, 2009; Munar et al., 2012a,b), in which participants are requested to decide whether the image they are viewing is beautiful or not beautiful. In this experiment, we expected (1) to corroborate the general tendency of lay participants to rate representational stimuli as beautiful and abstract ones as not beautiful, and (2) to find a longer RT when participants overrode the usual response (representational/beautiful, abstract/not beautiful). A second experiment using fMRI was designed to reveal brain activity related to these effects.

## Experiment 1

### Materials and methods

#### Participants

Twenty-six healthy participants, 14 women and 12 men, aged between 18 and 32 (*m* = 23.5 years, *SD* = 3.55), took part in the experiment. None of them had received training in art or art history. All participants had normal or corrected vision and normal color vision. The experiment was approved by the Ethical Committee of the *Comunitat Autònoma de les Illes Balears* (Spain), and all subjects participating in the study provided informed consent.

#### Materials and procedures

The stimuli consisted of 200 images selected from the stimuli pool used by Cela-Conde et al. (2004). These stimuli included photographs and reproductions of a variety of unfamiliar paintings by renowned artists. Participants viewed 100 abstract paintings and 100 representational paintings and photographs. All the stimuli had a resolution of 709 × 531 pixels and were used in.bmp format. The design was adapted from previous MEG studies (Cela-Conde et al., 2004, 2009) to suit an fMRI experiment (experiment 2).

**Figure 1:**
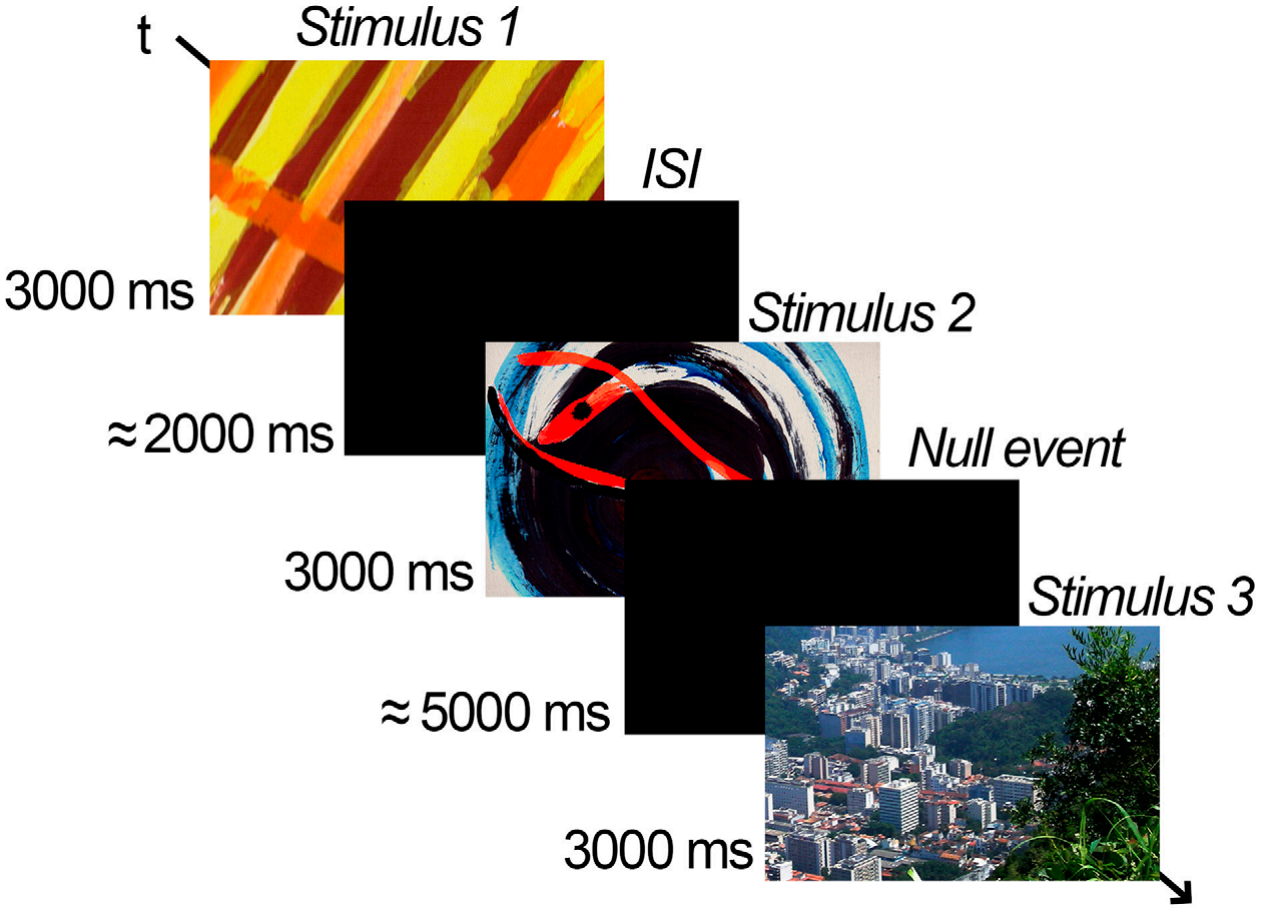
**Example of the experimental procedure**. A variable jitter time (ISI) of about 2 s followed or preceded a stimulus that could be an abstract painting, a representational painting, or a photograph, presented for 3 s. Participants were asked to decide whether the stimulus presented was beautiful or not. Additionally, null-events consisting of a black screen lasting the whole trial length (≈5 s) were included to establish a baseline in which no stimulus was presented and no response was given. Note: the examples provided here are by Gabriela de Oliveira, under license CC BY 4.0. For copyright reasons the actual stimuli used in the experiment could not be reproduced.

The session began with four practice and 200 test trials. Participants were asked to indicate whether they thought each stimulus was beautiful or not beautiful by pressing the B button or the N button on a computer keyboard. They were encouraged to respond with their forefingers. Every stimulus was presented for 3000 ms, followed by an interstimulus interval (black screen) of between 1900 and 2100 ms. Additionally, in order to allow the comparison with our subsequent fMRI experiment 2, in which reducing stimulus onset predictability and establishing a baseline is needed, 40 null-events consisting of a black screen lasting the whole trial length (≈5000 ms) were included. All trials and null events were randomized. Figure 1 illustrates the procedure.

Stimuli were presented using DirectRT, v.2006.2.0.28 (Empirisoft Corporation) running on a Windows XP SP3 PC (Intel Pentium Dual Core E5400, 2.70 GHz, 4GB RAM). They were displayed on a 19-inch screen (resolution: 1440 × 900 p; color: 32 bits; refresh rate: 60 Hz), which produced a black frame around the stimulus. The experiment was carried out in a well-lighted and suitable temperature computer laboratory, where participants were received in groups of 2–6 and were seated about 16 inches from the screen. Before the experiment began, participants were instructed that their task was to judge a series of images as beautiful or not beautiful. They were also told that they had a limited time to respond, and that the screen could remain black for a certain amount of time.

### Results

Two different analyses were carried out. First, we performed a standard repeated measures ANOVA to ascertain the impact of the kind of stimuli (abstract or representational) on esthetic appreciation (beautiful or not beautiful). Second, we carried out a specific analysis aimed at avoiding the influence of the number of stimuli in each category on the RT analysis. This analysis involved calculating a pooled beauty measure for every stimulus, based on the number of beautiful responses it received.

#### Repeated measures ANOVA

Regarding the number of responses awarded to each category, our results show that, on average, participants significantly rated more stimuli as not beautiful (*m* = 119.69, *SD* = 26.64) than as beautiful. (*m* = 77.46, *SD* = 27.43), *F*_(1, 25)_ = 15.902, *p* = 0.001, η^2^_p_ = 0.389.

The interaction effect between the kind of stimuli and the response was significant, *F*_(1, 25)_ = 51.093, *p* < 0.001, η^2^_p_ = 0.671. To break down this interaction, four contrasts were performed. All significant contrasts reported survived the Bonferroni correction for multiple comparisons. Within the abstract category, more stimuli were judged as not beautiful (*m* = 78.54, *SD* = 14.40) than beautiful (*m* = 20.00, *SD* = 14.37), *F*_(1, 25)_ = 107.893, *p* < 0.001, η^2^_p_ = 0.812. Although within the representational category, more stimuli were judged as beautiful (*m* = 57.46, *SD* = 22.94) than not beautiful (*m* = 41.15, *SD* = 22.45), this difference emerged only as a trend, *F*_(1, 25)_ = 3.361, *p* = 0.079, η^2^_p_ = 0.118. As for differences between stimuli kind within each response, our results show that most of the beautiful responses were awarded to representational stimuli compared to abstract stimuli, *F*_(1, 25)_ = 51.125, *p* < 0.001, η^2^_p_ = 0.672. Conversely, most of the not beautiful responses were awarded to abstract stimuli compared to representational stimuli, *F*_(1, 25)_ = 50.916, *p* < 0.001, η^2^_p_ = 0.671.

**Figure 2:**
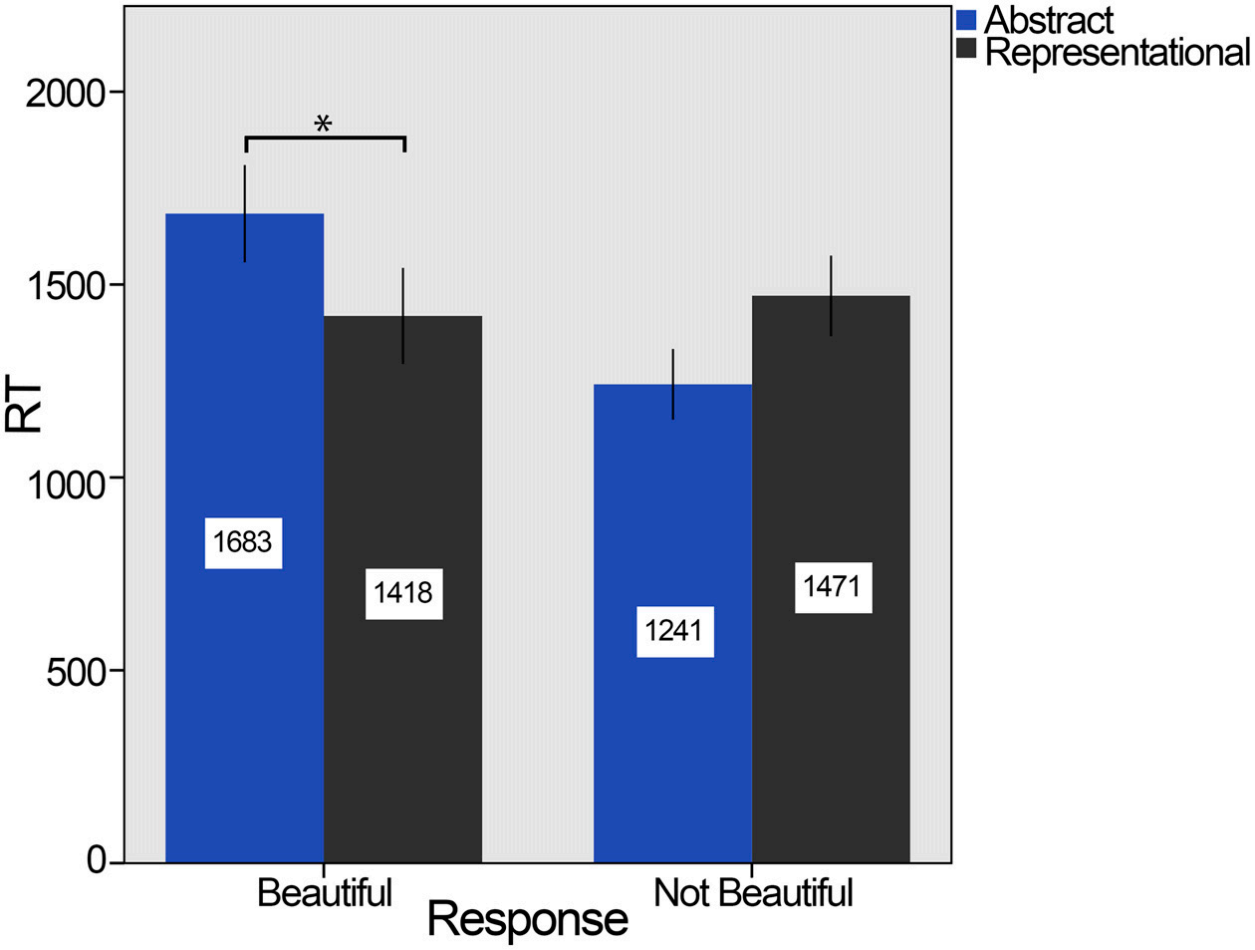
**Means of RTs in experiment 1**. Abstract stimuli (in blue) took significantly longer to be classified as beautiful than to be classified as not beautiful, while representational stimuli (in dark gray) show the reverse phenomenon but only as a trend. RT reported in milliseconds. The error bars refer to the 95% CI, confidence interval, and the ^*^ indicates significant difference *p* < 0.001.

Regarding RT, there was a significant main effect of the response. In general, stimuli took longer to be classified as beautiful (*m* = 1470 ms, *SD* = 280) than as not beautiful (*m* = 1310 ms, *SD* = 232), *F*_(1, 25)_ = 24.232, *p* = 0.001, η^2^_p_ = 0.492. However, there was no significant main effect of the kind of stimuli, *F*_(1, 25)_ = 0.286, *p* = 0.598, η^2^_p_ = 0.011: *m* = 1301 ms (*SD* = 217) for abstract and *m* = 1401 ms (*SD* = 264) for representational. However, the interaction effect between the kind of stimuli and the response was significant, *F*_(1, 25)_ = 62.065, *p* < 0.001, η^2^_p_ = 0.713. To break down this interaction, four contrasts were performed and all significant contrasts reported below survived the Bonferroni correction. For abstract stimuli, the beautiful ones took longer (*m* = 1683 ms, *SD* = 312 ms) than the not beautiful ones (*m* = 1241 ms, *SD* = 226) and this difference was significant [*F*_(1, 25)_ = 68.166, *p* < 0.001, η^2^_p_ = 0.732], while for representational stimuli the difference was not significant, although in this case the mean for not beautiful stimuli (*m* = 1471 ms, *SD* = 264) was only slightly higher than the mean for beautiful ones (*m* = 1418 ms, *SD* = 307) [*F*_(1, 25)_ = 1.219, *p* = 0.280, η^2^_p_ = 0.047]. On the other hand, the effect for the kind of stimuli was significant for beautiful [*F*_(1, 25)_ = 25.340, *p* < 0.001, η^2^_p_ = 0.503] and not beautiful responses [*F*_(1, 25)_ = 37.259, *p* < 0.001, η^2^_p_ = 0.598]. As shown in Figure 2, abstract stimuli took significantly longer to be classified as beautiful than to be classified as not beautiful. The reverse phenomenon is provided by representational stimuli that took longer to be classified as not beautiful.

#### Beauty proportion

It could be argued that longer RTs (abstract/beautiful and representational/not beautiful) are due to the lower frequency of these trials. This was the reason we carried out a second analysis based on the “beauty proportion” (used by Chatterjee et al., 2009). We calculated the proportion of “beautiful” answers across the participants for each stimulus. Thus, we obtained a score for every stimulus in a continuous scale from 0 (not beautiful) to 1 (beautiful). In this way, the correlation between the beauty proportion and the RT could be explored. This analysis attenuates the effect of frequency because it is based on the stimuli instead of participants. The correlation between RT and the beauty proportion of abstract stimuli was 0.58 (*p* < 0.001), and the correlation of RT and the beauty proportion of representational stimuli was −0.29 (*p* = 0.004). Additional analyses to palliate the effect of frequency on our results were performed and are included in the supplementary material.

### Discussion

As in previous studies, our lay participants generally considered abstract paintings to be not beautiful, and most of representational paintings as beautiful. These results support the notion of a general tendency to appreciate representational paintings as beautiful and abstract ones as not beautiful (“usual response” henceforth). This tendency can be understood as a consequence of familiarity and comprehensibility. Laypeople find representational art to be more familiar and understandable than abstract art.

The RT results from our experiment provide some indications on the processes in which an abstract painting is rated as beautiful and a representational one is rated as not beautiful. The abstract paintings took an average of 330 ms longer to be considered beautiful than not beautiful. On average, representational paintings took 190 ms less to be considered beautiful than not beautiful. It seems that some additional time is necessary to rate an abstract painting as beautiful and a representational one as not beautiful (“unusual response” henceforth) when comparing these trials with the usual response. We believe that this additional time implies more complex cognitive processing. This added complexity could reflect a competitive process between the usual response and higher cognitive information against this response. Esthetic appreciation is not only the outcome of a simple bottom-up perception, but several top-down cognitive resources could take part in this processing as Leder's et al. (2004) model indicated. Cultural influences, expectations, personal experience, repeated exposures, and attributions can influence esthetic appreciation. These influences could contradict the usual response and lead to an unusual one. Overriding the usual response, thus, seems to be the reason for the observed extended response times.

In the context of esthetic appreciation, high-level top-down processing and evaluative judgments have been related to activation of the left prefrontal cortex (PFC) (Cela-Conde et al., 2004; Jacobsen et al., 2006; Cupchik et al., 2009), the lateral orbitofrontal cortex (OFC) (Munar et al., 2012b) and the frontomedian cortex, the left temporal pole and the temporoparietal junction (Jacobsen et al., 2006). The monitorization of conflict resolution has been associated with activity in the anterior cingulate cortex (ACC) (Kawabata and Zeki, 2004; Vartanian and Goel, 2004; Cupchik et al., 2009; Kirk et al., 2009; Brown et al., 2011). Thus, we hypothesized that overriding usual esthetic responses to produce unusual esthetic responses involves activity in this network of cognitive control regions.

## Experiment 2

The objective of this experiment was to determine the neural mechanisms that underlie the longer processing for unusual responses. To achieve this we designed a neuroimaging study that would allow the comparison of brain activity, especially in the frontal lobes, while participants provide usual and unusual esthetic responses.

### Materials and methods

#### Participants

Twenty-four healthy participants, 12 women and 12 men, aged between 20 and 32 (*m* = 23.54 years, *SD* = 2.60), took part voluntarily. They were all undergraduate or graduate students at the University of Balearic Islands with no previous training in art or art history. They all had normal or corrected vision and normal color vision. None of them participated in experiment 1. The experiment was approved by the Ethical Committee of the *Comunitat Autònoma de les Illes Balears* (Spain), and all participants provided informed consent.

#### Materials and procedures

The design was the same as experiment 1, adapted to the fMRI session: participants lay supine in the scanner with head movements minimized by an adjustable padded head holder. Stimuli were presented on VisuaStim Digital Visor (Resonance Technology, Inc.) with a resolution of 800 × 600 pixels, which eliminated almost the entire black frame around the stimulus of experiment 1. Responses were recorded via an MR-compatible response grip. The participants were asked to indicate if they found each stimulus to be beautiful or not beautiful. They did so by pressing one button or another on the response grip with their forefingers. Half of the participants used the right forefinger to respond when they found the presented images to be beautiful, and the left forefinger to respond when they considered the image not beautiful; the other half used the left forefinger to indicate beautiful stimuli and the right one to indicate not beautiful ones. A PC running DirectRT v2006 (Empirisoft Corporation) on Windows XP controlled the stimulus presentation and response registration.

#### fMRI scanning and analysis

Images were acquired using a 1.5 Tesla scanner (Siemens MAGNETOM Symphony). Blood oxygenation level dependence (BOLD) sensitive functional images were acquired using a single shot gradient echo-planar imaging (EPI) sequence [*TR* (repetition time) = 2500 ms, *TE* (echo time) = 44 ms, *FOV* (field of view) = 192 mm, *FA* (flip angle) = 90°]. Each functional run consisted of 538 whole brain volumes comprising 25 transversal slices (voxel size 3 × 3 × 3 mm) axially aligned with a gap of 0.84 mm between them. The acquisition was interleaved bottom-up. An automatic shimming procedure was performed before each scanning session. Following the experimental session, structural images were acquired [sequence (*TR*/*TE*/*TI* (inversion time) 2140 ms/3.93 ms/1100 ms)] to include additional information on the normalizing step of the analysis.

Imaging data were analyzed using SPM8 (http://www.fil.ion.ucl.ac.uk/spm/software/spm8/; Wellcome Department of Imaging Neuroscience, London, UK), running on MatLab 7.9 (MathWorks, Inc.). The first six volumes of each functional run were discarded to allow for T1 equilibration (dummy scans). Pre-processing of the functional data included realignment, coregister of the structural images with the functional data, segment of the coregistered images to obtain the normalization parameters, normalization of the functional images and smoothing of them with an isotropic 8-mm full width-half-maximum Gaussian kernel.

An event-related factorial design was performed with two within-subject factors: kind of stimuli (abstract or representational) and participant response (beautiful or not beautiful). Movement parameters obtained in preprocessing were included as regressors in the design matrix. RT for each response was treated as a parametric modulator with a second order polynomial expansion. One bilateral region of interest (ROI) mask was created a priori to focus analyses to search for activity in frontal lobes. As noted above, unusual esthetic responses may involve activity in cognitive control regions, such as the PFC, OFC, frontomedian cortex, and ACC (see the Discussion of Experiment 1 for further details). To cover all these regions and other areas that may contribute to the overriding response, we focused on the full frontal lobes. In order to generate the mask, we used the WFU Pickatlas tool (Maldjian et al., 2003) in basic mode, including the left and right frontal lobes according to its Talairach Daemon labels. The factor effects at each voxel were estimated at group level according to the general linear model. Local and ROI effects were compared using linear contrasts. Each contrast produced a statistical parametric map of the *t* statistic for each voxel, which was subsequently transformed to a unit normal *z* distribution. One participant had to be excluded from the group analysis because first level model specification revealed technical problems during data acquisition.

### Results

#### Behavioral results

Analysis of the behavioral data revealed a significant difference in the number of responses of each class. Specifically, there were more stimuli classified as not beautiful (*m* = 111.83, *SD* = 23.19) than stimuli classified as beautiful (*m* = 85.29, *SD* = 23.30), *F*_(1, 23)_ = 7.937, *p* = 0.010, η^2^_p_ = 0.257. The ANOVA analysis showed a significant interaction effect between the kind of stimuli and the response, *F*_(1, 23)_ = 41.231, *p* < 0.001, η^2^_p_ = 0.642. To break down this interaction, four contrasts were performed: within the abstract category more stimuli were judged as not beautiful (*m* = 76.46, *SD* = 20.25) than beautiful (*m* = 22.00, *SD* = 18.75), *F*_(1, 23)_ = 47.083, *p* < 0.001, η^2^_p_ = 0.672. Within the representational category, more stimuli were judged as beautiful (*m* = 63.29, *SD* = 20.02) than not beautiful (*m* = 35.37, *SD* = 19.13), *F*_(1, 23)_ = 12.239, *p* = 0.002, η^2^_p_ = 0.347. As for differences between stimuli kind within each response, our results show that most of the beautiful responses were awarded to representational stimuli, as compared to abstract stimuli, *F*_(1, 23)_ = 42.519, *p* < 0.001, η^2^_p_ = 0.649. Conversely, most of the not beautiful responses were awarded to abstract stimuli, compared with representational stimuli. All contrasts survived the Bonferroni correction.

**Figure 3:**
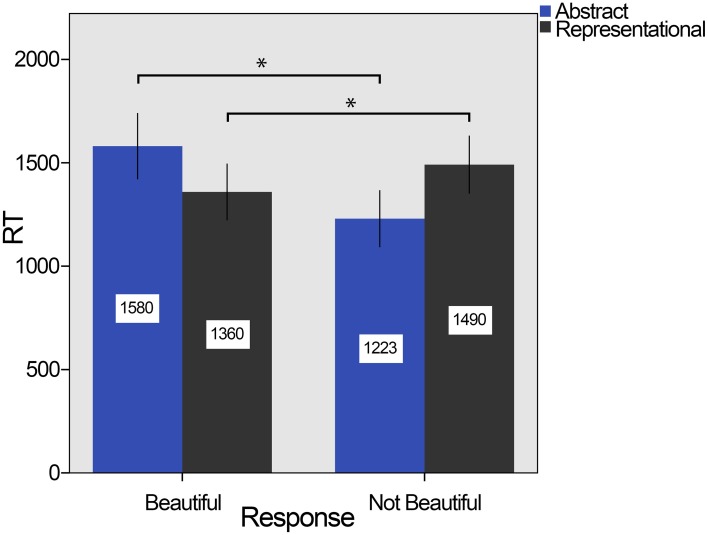
**Means of RT in experiment 2**. Abstract stimuli (in blue) took significantly longer to be classified as beautiful than to be classified as not beautiful, while representational stimuli (in dark gray) show the reverse phenomenon. RT reported in milliseconds. The error bars refer to 95% CI, confidence interval, and the ^*^ indicates significant difference *p* < 0.001.

Analysis of RTs revealed a significant main effect for the response: Stimuli took longer to be classified as beautiful (*m* = 1470 ms, *SD* = 315) than not beautiful [*m* = 1361 ms, *SD* = 316), *F*_(1, 23)_ = 5.443, *p* = 0.029, η^2^_p_ = 0.191. However, there was no main effect of the kind of stimuli, *F*_(1, 23)_ = 0.330, *p* = 0.571, η^2^_p_ = 0.014]: *m* = 1283 ms (*SD* = 352) for abstract and *m* = 1400 ms (*SD* = 336) for representational. There was, however, a significant interaction effect between the kind of stimuli and the response, *F*_(1, 23)_ = 37.014, *p* < 0.001, η^2^_p_ = 0.617. To break down this interaction, four contrasts were performed: the effect of response was significant for abstract [*F*_(1, 23)_ = 21.985, *p* < 0.001, η^2^_p_ = 0.489] and representational stimuli [*F*_(1, 23)_ = 9.003, *p* = 0.002, η^2^_p_ = 0.281], and the effect for the kind of stimuli was also significant for beautiful [*F*_(1, 23)_ = 11.747, *p* = 0.002, η^2^_p_ = 0.338] and not beautiful responses [*F*_(1, 23)_ = 48.727, *p* < 0.001, η^2^_p_ = 0.679]. Thus, abstract stimuli took less time to be judged as not beautiful (*m* = 1223 ms, *SD* = 330) than beautiful (*m* = 1580, *SD* = 380) and representational stimuli took less time to be judged as beautiful (*m* = 1360 ms, *SD* = 320) than not beautiful (*m* = 1490 ms, *SD* = 330). All reported contrasts survived the Bonferroni correction. These effects are shown in Figure 3.

***Beauty proportion correlation***. Following the analysis of experiment 1, a relative beauty score was calculated for each stimulus. Correlation of RT to beauty of abstract stimuli was 0.36 (*p* < 0.001). Correlation of RT to beauty of representational stimuli was −0.51 (*p* < 0.001).

#### fMRI results

An event-related factorial design was performed with two within-subject factors: kind of stimuli and participant response. Movement parameters obtained in preprocessing and RT for each response were included as regressors in the design matrix. Three *t*-test comparisons and their reverse counterparts were performed at first level. Two of them were exploratory, to reveal the activity related with abstraction and beauty. The last one was key to our main research question in this second experiment: whether there is different activity between unusual and usual responses in a visual esthetic preference task. One bilateral ROI mask was created *a priori* to focus analyses to search for activity in frontal lobes in order to test our hypothesis.

Table 1 displays the results of an exploratory analysis while taking into account the three contrasts with no ROI masking. To summarize, abstract stimuli were related to differential activity compared to representational ones in the cuneus and cingulate cortex. Conversely, representational stimuli were strongly related to occipito-temporal activity involving fusiform, parahippocampal, and lingual regions. Beautiful responses showed more activity than not beautiful ones mainly in the cingulate cortex and superior occipital gyrus. Reverse contrast showed no results. Unusual responses reveal different activity in a large frontal cluster with respect to usual responses. The reverse contrast showed activity in the suppramarginal gyri.

**Table 1:** **Effects of kind of stimulus and response (*t*-test)**.

**Contrast**	***p*-value adjustment**	**Region of activation (local maxima)**	**Brodmann area**	**Lat**	**Cluster size**	**Z-score**	**MNI coordinates**
Abstract > representational	<0.001 (uncorrected)^*^	Cuneus	18	B	142	4.69	3, −79, 31
		Middle cingulate	23	B	41	4.19	0, −19, 40
		Anterior cingulate	24	B	38	3.92	0, 26, 22
Representational > abstract	<0.05 (FDR-corrected)^*^	Fusiform—parahippocampal—lingual	37	B	43	6.31	−30, −43, −8
		Calcarine—precuneus	17–19	R	176	6.25	18, −55, 10
		Calcarine	30	L	17	4.96	−12, −52, 7
		Middle occipital—middle temporal	39	B	133	6.28	45, −76, 19
					102	6.00	−39, −79, 25
Beautiful > not beautiful	<0.001 (uncorrected)	Subthalamic nucleus	–	R	18	4.03	15, −13, −8
		Anterior-middle cingulate	24	L	36	4.00	−3, 5, 28
		Inferior parietal	40	L	12	3.93	−33, −34, 37
		Superior occipital	7	R	30	3.65	27, −64, 43
Beautiful > not beautiful	*ns*						
Unusual > usual resp	<0.001 (uncorrected)^*^	Dorsal anterior cingulate—superior medial frontal—supplementary motor area	32, 8, 9, 24	B	452	4.59	3, 29, 37
		Middle frontal	9–10	R	44	4.40	21, 47, 28
Usual > unusual resp	<0.001 (uncorrected)^*^	Suppramarginal—postcentral—superior temporal	42, 48, 22	R	36	4.46	63, −22, 19
		Suppramarginal	48	L	30	4.22	−66, −34, 25

p-value adjustment refers to peak-level. An

* *indicates that activations reported survived the FWE correction at cluster level (p < 0.05). Cluster size in voxels. “Lat” indicates laterality (R, right; L, left; B, bilateral). Degrees of freedom = [1.0, 22.0]*.

**Figure 4:**
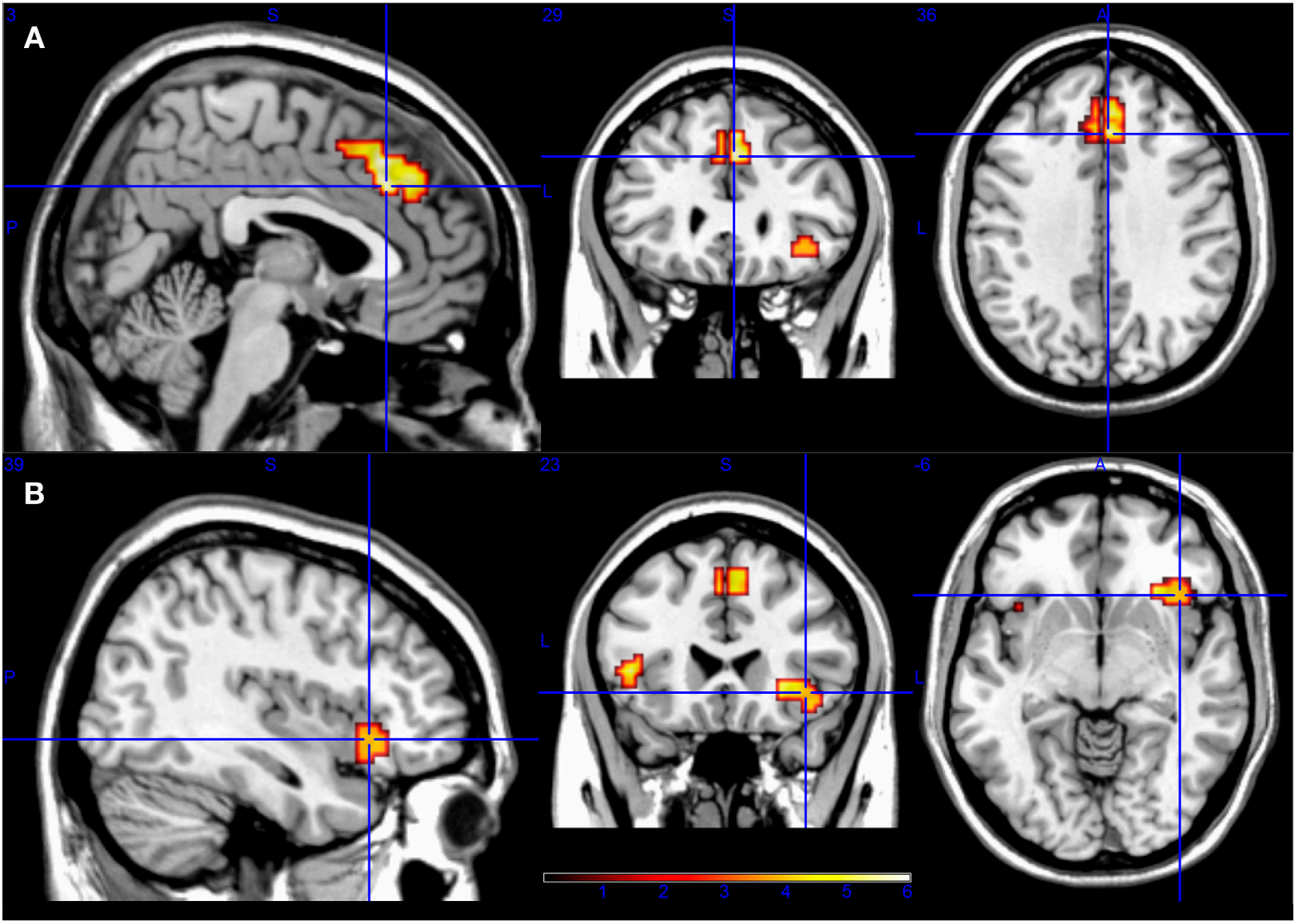
**ROI analysis results for unusual-vs.-usual contrast**. It indicates that frontal lobes are related to the overcoming of usual responses, involving anterior cingulate cortex, superior frontal gyrus, and supplementary motor area **(A)**; and also right orbitofrontal cortex and bilateral insula **(B)**.

The only contrast that still produced significant results with the ROI masking was the unusual-vs.-usual response one. The main differential activity was a large cluster (*k* = 144 voxels) which local maximum is in the dorsal ACC (MNI coordinates: 3, 29, 37; *p* < 0.001 uncorrected). It extended to the superior frontal gyrus and the supplementary motor area. Additionally, another smaller cluster (*k* = 34) was found in the right OFC and activity extended bilaterally to the anterior insula. Figure 4 illustrates these results.

### Discussion

Our behavioral results confirmed what other previous experimental studies showed: laypeople esthetically appreciate representational paintings more than abstract ones (Hekkert and van Wieringen, 1996; Furnham and Walker, 2001a; Cusack et al., 2010; Nadal et al., 2010; Vessel and Rubin, 2010; Pihko et al., 2011). Significant differences appeared in our two experiments and the data reanalysis from Nadal et al. (2010). What previous studies did not report is that unusual responses take a significantly longer time than usual responses.

The comparison of frontal activity in usual and unusual responses revealed that the unusual responses showed a higher activity in two clusters. The main cluster had focus in the dorsal ACC extending to superior frontal gyrus and supplementary motor area. The smaller cluster was focused in the right OFC extending to the anterior insula.

The ACC has been related to a wide variety of roles. Two major subdivisions seem to subserve distinct functions (Bush et al., 2000; Paus, 2001): a dorsal cognitive division and a rostral-ventral affective division. The dorsal division has reciprocal connections with PFC. The main cluster of our fMRI results was focused on the dorsal division, and the other active cluster was located in prefrontal areas (OFC and insula). Moreover, this consideration was supported by the presence of a robust functional connectivity between the supracallosal ACC and the PFC, unlike the subcallosal ACC. Some studies suggest that both dorsal ACC and lateral PFC areas act together during tasks that involve high levels of mental effort (Dehaene et al., 1998; Bush et al., 2000). These areas operate in a common influence during many diverse mental processes (Duncan and Owen, 2000). More specifically, it could be considered that the change from usual to unusual response involves conflict monitoring. In this line, it was suggested that the role of the ACC is related to conflict monitoring and error detection (Pardo et al., 1990; Brown and Braver, 2005; Carter and van Veen, 2007). However, some studies provide evidence that the ACC does not have an indispensable role in conflict-induced behavioral adjustment and calls this assumption into question (Mansouri et al., 2007, 2009).

Walton et al. (2007) proposed that the ACC plays a prominent role within a distributed network which determines the dynamic value of actions and guide decision making appropriately. They considered the ACC's role in more naturalistic situations where there is no single certain correct response and the relationships between choices and their consequences vary, which are similar circumstances to our experimental task. Walton and Mars (2007) suggested that the ACC is important for interpreting outcome information according to the current task context to guide future action selection. In this line, two complementary valuation processes have been proposed (Kennerley et al., 2011; Wallis and Kennerley, 2011): the OFC would encode the value of choice relative to the value context recently experienced, while the ACC would encode choice predictions using a common valuation currency reflecting the integration of multiple decision parameters. The ACC would evaluate the consequences of the choice and could play an important role in learning and enabling the modification of future choices. We interpret our results in this way, with a main cluster in the ACC and the other one in the OFC. The higher activity in the ACC would mean the need for intense processing to integrate more parameters to go against the usual response and this activity would not be necessary during a more usual response.

Previous studies on the neural correlates of esthetic appreciation have found activity in the ACC related to the esthetic valuation (Kawabata and Zeki, 2004; Vartanian and Goel, 2004; Kirk et al., 2009). However, all of them found the activity in the subcallosal region of the ACC which is connected mostly to some limbic areas related to the second division indicated by Bush et al. (2000): the rostral-ventral affective division. For this reason, these three works mainly related the ACC activation to emotional valence and reward properties. Brown et al. (2011) performed a meta-analysis of neuroesthetic processing based on 93 neuroimaging studies across four sensory modalities. They proposed a model in which esthetic appraisal is seen as a comparison between extereoceptive information (OFC) and interoceptive information (anterior insula), and the most pregenual anterior cingulate (ACC) is involved in “emotional salience monitoring.” Our fMRI results showed similar activated areas as those in other neuroaesthetic studies and these areas are the main ones in Brown's model. However, as the ACC activation was more dorsal than ventral in our results, we consider that this activation reflects a processing increase of parameters to make a more intense analysis that leads to a response that goes against the more usual one.

If our interpretation of these results is correct, a different effect could be expected in art experts whose knowledge and familiarity with abstract art could modulate their responses and RT. Laypeople's appreciation of art is intrinsically related to recognition of the depicted elements. Whereas experts focus more on background features, composition, and color contrasts, untrained viewers, such as the ones participating in this study, focus more on individual figurative elements and exploring figures in the center and foreground (Winston and Cupchik, 1992; Nodine et al., 1993; Hekkert and van Wieringen, 1996). Given that the cues on which laypeople rely for their appreciation are, by definition, present in representational stimuli and absent in abstract stimuli, the added effort to judge a representational stimulus as not beautiful might actually involve different underlying processes than the added effort to judge an abstract stimulus as beautiful. In the former case, such added processes are probably related to the semantic content of the depicted elements, while in the latter case, they are probably related to purely formal aspects, such as color, form, texture, and so on. Further studies are required to clarify this matter. On the other hand, the additional time effect seems to be stronger in abstract than representational stimuli, possibly due to greater semantic (even emotional) content of representational stimuli. This also is a pending empirical issue for future research.

### Conflict of interest statement

The authors declare that the research was conducted in the absence of any commercial or financial relationships that could be construed as a potential conflict of interest.

## References

[B1] Brown J. W. Braver T. S. (2005). Learned predictions of error likelihood in the anterior cingulate cortex. Science 307, 1118–1121 10.1126/science.1105783 15718473

[B2] Brown S. Gao X. Tisdelle L. Eickhoff S. B. Liotti M. (2011). Naturalizing aesthetics: brain areas for aesthetic appraisal across sensory modalities. Neuroimage 58, 250–258 10.1016/j.neuroimage.2011.06.012 21699987 PMC8005853

[B3] Bush G. Luu P. Posner M. I. (2000). Cognitive and emotional influences in anterior cingulate cortex. Trends Cogn. Sci. 4, 215–222 10.1016/S1364-6613(00)01483-2 10827444

[B4] Cantor G. N. Kubose S. K. (1969). Preschool children's ratings of familiarized and nonfamiliarized visual stimuli. J. Exp. Child Psychol. 8, 74–81 10.1016/0022-0965(69)90029-0 5804593

[B5] Carter C. S. van Veen V. (2007). Anterior cingulate cortex and conflict detection: an update of theory and data. Cogn. Affect. Behav. Neurosci. 7, 367–379 10.3758/CABN.7.4.367 18189010

[B6] Cela-Conde C. J. Ayala F. J. Munar E. Maestú F. Nadal M. Capó M. A. (2009). Sex-related similarities and differences in the neural correlates of beauty. Proc. Natl. Acad. Sci. U.S.A. 106, 3847–3852 10.1073/pnas.0900304106 19237562 PMC2656168

[B7] Cela-Conde C. J. Marty G. Maestú F. Ortiz T. Munar E. Fernandez A. (2004). Activation of the prefrontal cortex in the human visual aesthetic perception. Proc. Natl. Acad. Sci. U.S.A. 101, 6321–6325 10.1073/pnas.0401427101 15079079 PMC395967

[B8] Cela-Conde C. J. Marty G. Munar E. Nadal M. Burges L. (2002). The “style scheme” grounds perception of paintings. Percept. Mot. Skills 95, 91–100 10.2466/pms.2002.95.1.91 12365279

[B9] Chatterjee A. Thomas A. Smith S. E. Aguirre G. K. (2009). The neural response to facial attractiveness. Neuropsychology 23, 135–143 10.1037/a0014430 19254086

[B10] Cupchik G. C. Vartanian O. Crawley A. Mikulis D. J. (2009). Viewing artworks: contributions of cognitive control and perceptual facilitation to aesthetic experience. Brain Cogn. 70, 84–91 10.1016/j.bandc.2009.01.003 19223099

[B11] Cusack P. Lankston L. Isles C. (2010). Impact of visual art in patient waiting rooms: survey of patients attending a transplant clinic in Dumfries. JRSM Short Rep. 1, 52 10.1258/shorts.2010.010077 21234115 PMC2994357

[B12] Dehaene S. Kerszberg M. Changeux J. P. (1998). A neuronal model of a global workspace in effortful cognitive tasks. Proc. Natl. Acad. Sci. U.S.A. 95, 14529–14534 10.1073/pnas.95.24.14529 9826734 PMC24407

[B13] Duncan J. Owen A. M. (2000). Common regions of the human frontal lobe recruited by diverse cognitive demands. Trends Neurosci. 23, 475–483 10.1016/S0166-2236(00)01633-7 11006464

[B14] Faw T. T. Nunnally J. C. (1971). The influence of stimulus incongruity on the familiarity effect in visual selection. Percept. Psychophys. 9, 150–154 10.3758/BF03212618

[B15] Furnham A. Walker J. (2001a). Personality and judgements of abstract, pop art, and representational paintings. Eur. J. Pers. 15, 57–72 10.1002/per.340

[B16] Furnham A. Walker J. (2001b). The influence of personality traits, previous experience of art, and demographic variables on artistic preference. Pers. Individ. Dif. 31, 997–1017 10.1016/S0191-8869(00)00202-6

[B17] Hargreaves D. J. (1984). The effects of repetition on liking for music. J. Res. Music Educ. 32, 35–47 10.2307/3345279

[B18] Hekkert P. van Wieringen P. (1996). The impact of level of expertise on the evaluation of original and altered versions of post-impressionistic paintings. Acta Psychol. 94, 117–131 10.1016/0001-6918(95)00055-0

[B19] Jacobsen T. Schubotz R. I. Höfel L. Cramon D. Y. V. (2006). Brain correlates of aesthetic judgment of beauty. Neuroimage 29, 276–285 10.1016/j.neuroimage.2005.07.010 16087351

[B20] Kawabata H. Zeki S. (2004). Neural correlates of beauty. J. Neurophysiol. 91, 1699–1705 10.1152/jn.00696.2003 15010496

[B21] Kennerley S. W. Behrens T. E. J. Wallis J. D. (2011). Double dissociation of value computations in orbitofrontal and anterior cingulate neurons. Nat. Neurosci. 14, 1581–1589 10.1038/nn.2961 22037498 PMC3225689

[B22] Kirk U. Skov M. Hulme O. Christensen M. S. Zeki S. (2009). Modulation of aesthetic value by semantic context: an fMRI study. Neuroimage 44, 1125–1132 10.1016/j.neuroimage.2008.10.009 19010423

[B23] Leder H. Belke B. Oeberst A. Augustin D. (2004). A model of aesthetic appreciation and aesthetic judgments. Br. J. Psychol. 95(Pt 4), 489–508 10.1348/0007126042369811 15527534

[B24] Maldjian J. A. Laurienti P. J. Kraft R. A. Burdette J. H. (2003). An automated method for neuroanatomic and cytoarchitectonic atlas-based interrogation of fMRI data sets. Neuroimage 19, 1233–1239 10.1016/S1053-8119(03)00169-1 12880848

[B25] Mansouri F. A. Buckley M. J. Tanaka K. (2007). Mnemonic function of the dorsolateral prefrontal cortex in conflict-induced behavioral adjustment. Science 318, 987–990 10.1126/science.1146384 17962523

[B26] Mansouri F. A. Tanaka K. Buckley M. J. (2009). Conflict-induced behavioural adjustment: a clue to the executive functions of the prefrontal cortex. Nat. Rev. Neurosci. 10, 141–152 10.1038/nrn2538 19153577

[B27] Monin B. (2003). The warm glow heuristic: when liking leads to familiarity. J. Pers. Soc. Psychol. 85, 1035 10.1037/0022-3514.85.6.1035 14674812

[B28] Munar E. Nadal M. Castellanos N. P. Flexas A. Maestú F. Mirasso C. (2012a). Aesthetic appreciation: event-related field and time-frequency analyses. Front. Hum. Neurosci. 5:185 10.3389/fnhum.2011.00185 22287948 PMC3251833

[B29] Munar E. Nadal M. Rosselló J. Flexas A. Moratti S. Maestú F. (2012b). Lateral orbitofrontal cortex involvement in initial negative aesthetic impression formation. PLoS ONE 7:e38152 10.1371/journal.pone.0038152 22675517 PMC3367021

[B30] Nadal M. Munar E. Marty G. Cela-Conde C. J. (2010). Visual complexity and beauty appreciation: explaining the divergence of results. Empirical Stud Arts 28, 173–191 10.2190/EM.28.2.d

[B31] Nodine C. F. Locher P. J. Krupinski E. A. (1993). The role of formal art training on perception and aesthetic judgment of art compositions. Leonardo 26, 219–227 10.2307/1575815

[B32] Pardo J. Pardo P. Janer K. Raichle M. (1990). The anterior cingulate cortex mediates processing selection in the stroop attentional conflict paradigm. Proc. Natl. Acad. Sci. U.S.A. 87, 256–259 10.1073/pnas.87.1.256 2296583 PMC53241

[B33] Paus T. (2001). Primate anterior cingulate cortex: where motor control, drive and cognition interface. Nat. Rev. Neurosci. 2, 417–424 10.1038/35077500 11389475

[B34] Pihko E. Virtanen A. Saarinen V.-M. Pannasch S. Hirvenkari L. Tossavainen T. (2011). Experiencing art: the influence of expertise and painting abstraction level. Front. Hum. Neurosci. 5:94 10.3389/fnhum.2011.00094 21941475 PMC3170917

[B35] Tinio P. P. L. Leder H. (2009). Just how stable are stable aesthetic features? Symmetry, complexity, and the jaws of massive familiarization. Acta Psychol. 130, 241–250 10.1016/j.actpsy.2009.01.001 19217589

[B36] Vartanian O. Goel V. (2004). Neuroanatomical correlates of aesthetic preference for paintings. Neuroreport 15, 893–897 10.1097/00001756-200404090-00032 15073538

[B37] Vessel E. A. Rubin N. (2010). Beauty and the beholder: highly individual taste for abstract, but not real-world images. J. Vis. 10, 18, 1–14. 10.1167/10.2.18 20462319 PMC3662030

[B38] Wallis J. D. Kennerley S. W. (2011). Contrasting reward signals in the orbitofrontal cortex and anterior cingulate cortex. Ann. N.Y. Acad. Sci. 1239, 33–42 10.1111/j.1749-6632.2011.06277.x 22145873

[B39] Walton M. E. Croxson P. L. Behrens T. E. J. Kennerley S. W. Rushworth M. F. S. (2007). Adaptive decision making and value in the anterior cingulate cortex. Neuroimage 36, T142–T154 10.1016/j.neuroimage.2007.03.029 17499161 PMC2954047

[B40] Walton M. E. Mars R. B. (2007). Probing human and monkey anterior cingulate cortex in variable environments. Cogn. Affect. Behav. Neurosci. 7, 413–422 10.3758/CABN.7.4.413 18189014 PMC2519031

[B41] Winston A. S. Cupchik G. C. (1992). The evaluation of high art and popular art by naive and experienced viewers. Visual Arts Res. 1–14

[B42] Zajonc R. B. (1968). Attitudinal effects of mere exposure. J. Pers. Soc. Psychol. 9(2p2), 1 5667435

[B43] Zizak D. M. Reber A. S. (2004). Implicit preferences: The role(s) of familiarity in the structural mere exposure effect. Conscious. Cogn. 13, 336–362 10.1016/j.concog.2003.12.003 15134764

